# Functional Flexibility of Exosomes and MicroRNAs of Intestinal Epithelial Cells in Affecting Inflammation

**DOI:** 10.3389/fmolb.2022.854487

**Published:** 2022-05-11

**Authors:** Eun Jeong Park, Motomu Shimaoka, Hiroshi Kiyono

**Affiliations:** ^1^ Department of Molecular Pathobiology and Cell Adhesion Biology, Mie University Graduate School of Medicine, Tsu, Japan; ^2^ Department of Mucosal Immunology, IMSUT Distinguished Professor Unit, The Institute of Medical Science, The University of Tokyo, Tokyo, Japan; ^3^ Division of Mucosal Vaccines, International Research and Development Center for Mucosal Vaccines, The Institute of Medical Science, The University of Tokyo, Tokyo, Japan; ^4^ Mucosal Immunology and Allergy Therapeutics, Institute for Global Prominent Research, Future Medicine Education and Research Organization, Chiba University, Chiba, Japan; ^5^ CU-UCSD Center for Mucosal Immunology, Allergy, and Vaccine (cMAV), Division of Gastroenterology, Department of Medicine, University of California, San Diego, CA, United States

**Keywords:** intestinal epithelial cell, lymphocyte, exosome, miRNA, sepsis, aging, inflammation, tight junction

## Abstract

Intestinal epithelial cells (IECs) are a mucosal immune barrier essential to coordinate host–microbe crosstalk. Sepsis is a systemic inflammatory syndrome with dysfunction in multiple organs including the intestine whose epithelial barrier is deregulated. Thus, IECs are a main contributor to intestinal permeability and inflammation in sepsis. Exosomes emerge as a mediator of intercellular and inter-organic communications. Recently, IEC-derived exosomes and their cargoes, such as microRNAs (miRNAs), in sepsis were shown to regulate the expression of proinflammatory mediators in the inflamed gut tissues. It is a compelling hypothesis that these IEC exosomes exhibit their dynamic activity to deliver their functional miRNA cargoes to immune cells in local and distant organs to regulate proinflammatory responses and alleviate tissue injury. Also, epithelial tight junction (TJ) proteins are downregulated on gut inflammation. Some of the IEC miRNAs were reported to deteriorate the epithelial integrity by diminishing TJ expressions in intestines during sepsis and aging. Thus, it is worth revisiting and discussing the diverse functions of IEC exosomes and miRNAs in reshaping inflammations. This review includes both iterative and hypothetical statements based on current knowledge in this field.

## Introduction

Sepsis is a serious illness of multiple organ dysfunction caused by systemic infection and dysregulated immune responses (Singer et al., 2016). Almost 49 million people are affected by sepsis every year, and among them, the mortality of approximately 22% corresponding to 11 million deaths is estimated (Rudd et al., 2020). The intestine is an organ severely damaged by sepsis, and the compromised epithelial barrier function represents a sepsis-induced pathologic defect (Yoseph et al., 2016). Along with such physical alterations, proinflammatory mediators secreted by intestines during sepsis are thought to be released into blood circulation, which may reach distant organs, including the lungs or liver, and contribute to aggravating tissue inflammation in multiple organs (Hack et al., 1997; Astiz and Rackow, 1998).

The intestinal epithelial cells (IECs) composed of a monolayer of polarized cells work as an elaborate coordinator of mucosal immune responses to foreign microbes (Allaire et al., 2018). The surface area of human IECs apically facing the foreign environment estimates approximately 32 m^2^, which corresponds to a half of a badminton court (Helander and Fandriks, 2014). Therefore, IECs may be defined as “the largest system of secreting diverse mediators, aside from their fundamental roles in driving nutrition absorption and pathogenic protection”.

Exosomes are nanosized extracellular vesicles and play a role in mediating intercellular communication *via* transfer of their biological cargoes such as proteins, messenger RNAs (mRNAs), or microRNAs (miRNAs). Consequently, source cell-derived exosomes alter target-cell functions to thereby remodel microenvironmental niches in target tissues. The exosomes secreted from different organs including the lungs, liver, pancreas, or intestine during sepsis are thought to systemically traffic to undergo intertissue crosstalk and induce septic pathogenesis and inflammation in distant tissues. During pancreatitis, pancreas-released exosomes enter into the liver to elicit hepatic cells to secrete functional exosomes, which consequently reach alveolar tissues to spread inflammation by activating lung macrophages (Bonjoch et al., 2016). Septic exosomes might play a role in disseminating inflammation and possibly devastating, or even attenuating, tissue damages *via* intertissue communications (Park et al., 2019a). Serum exosomes isolated from septic mice were shown to exert protective effects on the diminishing expression of proinflammatory mediators in serum and organs (such as the lung, kidney, and liver) and alleviating pathohistological grades in those septic tissues (Li et al., 2021). Accordingly, the effect of the exosomes on affecting inflammation might be diversified depending, at least partly, on their source organs and cells.

The miRNAs, which are single-stranded non-coding RNA molecules, fine-tune cellular function by restricting the target-gene expression within target cells *via* post-transcriptional modifications (Kim and Nam, 2006; O’connell et al., 2010). The miRNAs bind, *via* their complementary seed sequences (6–8 nucleotides in length), to the 3′-untranslated region (3′-UTR) of target genes and repress their expressions. The miRNAs present within cells and exosomes are capable of affecting the expression of specific genes inside target cells and modifying their functions. Thus, it will be of great importance to examine 1) how cells and exosomes alter their expression and composition of functional miRNAs, depending on the progressions of sepsis and aging and 2) how those modifications of cells and exosomes regulate pathologic progressions of systemic inflammation.

It is unquestionable that sepsis is a syndrome detrimental to people of all ages. Nonetheless, the elderly patients with sepsis are much more vulnerable to morbidity and complications and show 1.3- to 1.5-times higher mortality than younger patients (Martin et al., 2017). Accordingly, it might be of significance to determine the function of the IEC miRNAs, and their gene regulation profiles and networks prominently emerged with age. This review discusses about the roles played by exosomes and miRNAs of IECs in modulating sepsis- and aging-associated inflammation. We also overviewed the effects of miRNAs on targeting epithelial TJ molecules and consequently eliciting deterioration or amelioration of inflammation. Although a large number of research studies relevant to the current review have been reported, only some of those previous studies were chosen by our present interest due to the restricted space. We, thus, would like to apologize that we were not able to cite many original and important works in this article.

### Possible Roles of Apically Released Intestinal Epithelial Cell Exosomes in Attenuating Gut Inflammation in Sepsis

The issue on how septic exosomes and their miRNAs participate in reshaping inflammatory progression remains controversial. Platelet-derived circulating exosomes in sepsis are capable of causing myocardial dysfunction and endothelial apoptosis (Azevedo et al., 2007; Gambim et al., 2007). Moreover, those platelet exosomes enhance neutrophil extracellular trap (NET) formation to aggravate sepsis-induced organ injury through a mechanism by which exosomal miR-15b-5p and miR-378a-3p target phosphoinositide-dependent protein kinase 1 (PDK1) (Jiao et al., 2020). In contrast, mesenchymal stem cell (MSC)-derived exosomes play a role in attenuating, *via* their miR-223, heart damages in sepsis (Wang et al., 2015). In addition, endothelial progenitor cell-derived exosomes seem to ameliorate lung injury in sepsis through miR-126’s post-transcriptional regulation of key targets, such as Sprouty-related protein with an EVH1 domain (SPRED-1), high mobility group box 1 (HMGB1), and vascular cell adhesion molecule 1 (VCAM1) (Wu et al., 2018; Zhou et al., 2018; Zhou et al., 2019). In contrast, polymorphonuclear cell-derived exosomes devastate lung alveolar injury in chronic pulmonary inflammation (Genschmer et al., 2019). Accordingly, sepsis-induced inflammatory tissue injury and multiple organ failure may produce a variety of functionally altered exosomes released from multiple sources, although these effects of exosomes might be differed at least partly depending on source cells.

Luminal IEC exosomes increased, following *Cryptosporidium parvum* infection, and were shown to activate toll-like receptor 4 (TLR4) signaling, which further mediate epithelial exosomes to shuttle antimicrobial peptides and thus attenuate *C. parvum* infectivity (Hu et al., 2013). Activation of TLR4 signaling was revealed to downregulate the expression of the let-7 family, which coupled to an increase in synaptosome-associated protein 23 (SNAP23) and a promotion of exosomal secretion of IECs (Hu et al., 2013). Tong et al. have shown that IEC-derived miR-146a-5p aggravates intestinal damage during sepsis using a model of rats (Tong et al., 2020). In this study, miR-146a-5p–mediated reduction in expressions of Kruppel-like factor 4 (Klf4) and cyclin D2 were thought to inhibit IEC proliferation and worsen gut injury on sepsis, although the role of the septic IEC exosome-derived miR-146b-5p remains unknown. Mitsuhashi et al. have observed that luminal aspirate-derived exosomes of inflammatory bowel disease (IBD) patients exhibit an increase in proinflammatory cytokines, such as interleukin 6 (IL-6), IL-8, and tumor necrosis factor α (TNF-α), compared to those of healthy volunteers (Mitsuhashi et al., 2016). These IBD exosomes were shown to promote macrophage migration *in vitro*, reflecting their increased traits of the proinflammatory activity (Mitsuhashi et al., 2016). Related to these notes, it will be imperative to probe any functional and expressional changes in aspirate exosomal miRNAs during inflammation.

**FIGURE 1:**
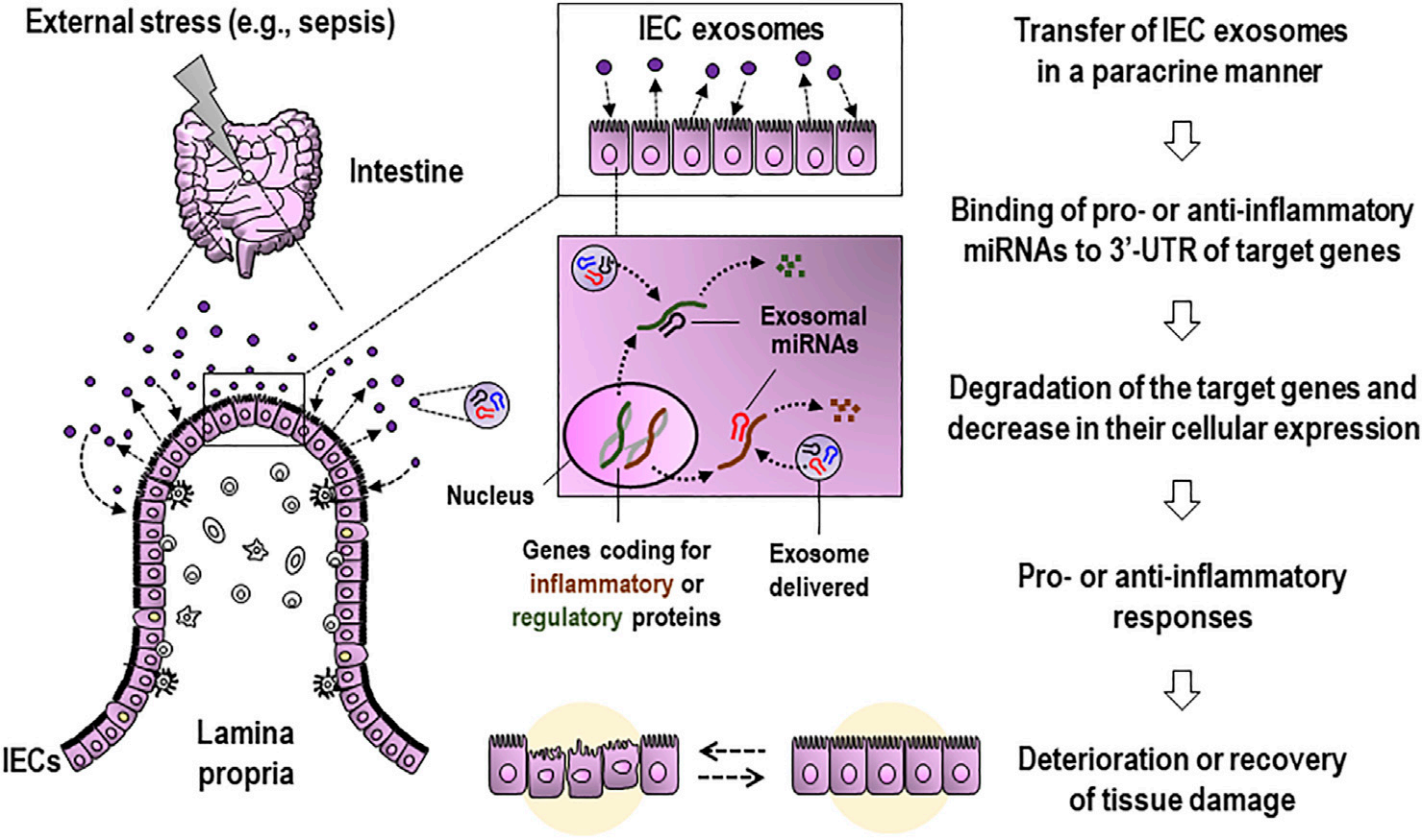
Proposed model for the role of apically released IEC exosomes during external stress, such as sepsis, in affecting tissue damage and gut inflammation. Luminally secreted IEC exosomes reach neighboring cells in a paracrine manner and transfer functional miRNAs to the cells. Then, the IEC-derived miRNAs are expected to be capable of suppressing the gene expression of pro- or anti-inflammatory mediators and proteins at post-transcriptional levels and consequently alleviating or aggravating inflammation-associated intestinal injury and barrier dysfunction. IEC, intestinal epithelial cell; 3′-UTR, 3′-untranslated region.

As distinct from the issue about contrasting effects of exosomes, probing activities of the exosomes present in biological samples remain a technical challenge, especially in isolating single origin-derived exosomes from liquid biopsies such as blood or bronchoalveolar lavage fluid. It is critical to uncover the roles played by sepsis-induced exosomes and their miRNAs, which are derived from identified origin, in aggravation or amelioration of septic pathology. A majority of the exosomes isolated from the intestinal-lavage fluid in sepsis were shown to be IEC-derived and appeared to contain miRNAs exhibiting the anti-inflammatory activity to downregulate the expression of proinflammatory mediators, including TNF-α and IL-17A (Appiah et al., 2020). These luminally released exosomes from IECs are thought to reach neighboring cells back in a paracrine manner and transfer miRNAs, including miR-19a, miR-21a, miR-27a, and miR-126a, functional in negatively regulating the expression of proinflammatory cytokines (Appiah et al., 2020). Consequently, these findings suggest that IEC exosomes can potentially alter inflammatory symptoms *via* their bioactive contents, including miRNAs, implying the functional variety of these exosomes and their cargoes. Figure 1 illustrates the proposed model for the potential roles of apically secreted IEC exosomes in affecting tissue damage and gut inflammation, in which their functional miRNAs contribute to regulating expressions of key molecules.

### Possible Roles of Basally Released Septic Intestinal Epithelial Cell Exosomes in Regulating Systemic Inflammation in Local and Distant Tissues

Exosomes possess dynamic properties to undergo intertissue traffic *via* systemic circulation and home to target organs, which is often mediated by interaction of integrins and their cognate ligands as do their source cells (Yanez-Mo et al., 2015; Park et al., 2020). The inter-organ migration played by exosomes was conducted to deliver their cargoes and remodel target tissue niches in an integrin-dependent manner (Myint et al., 2020). Breast cancer exosomes were found to reach the lung tissue *via* α6β4- fibronectin and liver tissue *via* αVβ5-laminin interactions to activate Src kinase and elicit a proinflammatory trait to form premetastatic niches (Hoshino et al., 2015). In addition, T-cell exosomes expressing α4β7 were shown to contain the gut-tropic property and downregulating the endothelial expression of the main α4β7 ligand mucosal addressin cell adhesion molecule 1 (MAdCAM-1) (Park et al., 2019a). The functional miRNAs contained in α4β7-positive T-cell exosomes may play a role in regulating the *MAdCAM-1* expression in the small intestine (Park et al., 2019b). The findings obtained in these previous reports suggest that IEC exosomes may remodel microenvironmental niches in both local and distant organs. Involvement of integrins in the migration of IEC exosomes to different organs remains a mystery, which might be a fascinating issue. Following this section, we discussed possible roles played by IEC exosomes in distant organs with the exclusion of integrin’s possible engagement but with focusing on passive migration to distant organs *via* systemic dissemination.

**FIGURE 2:**
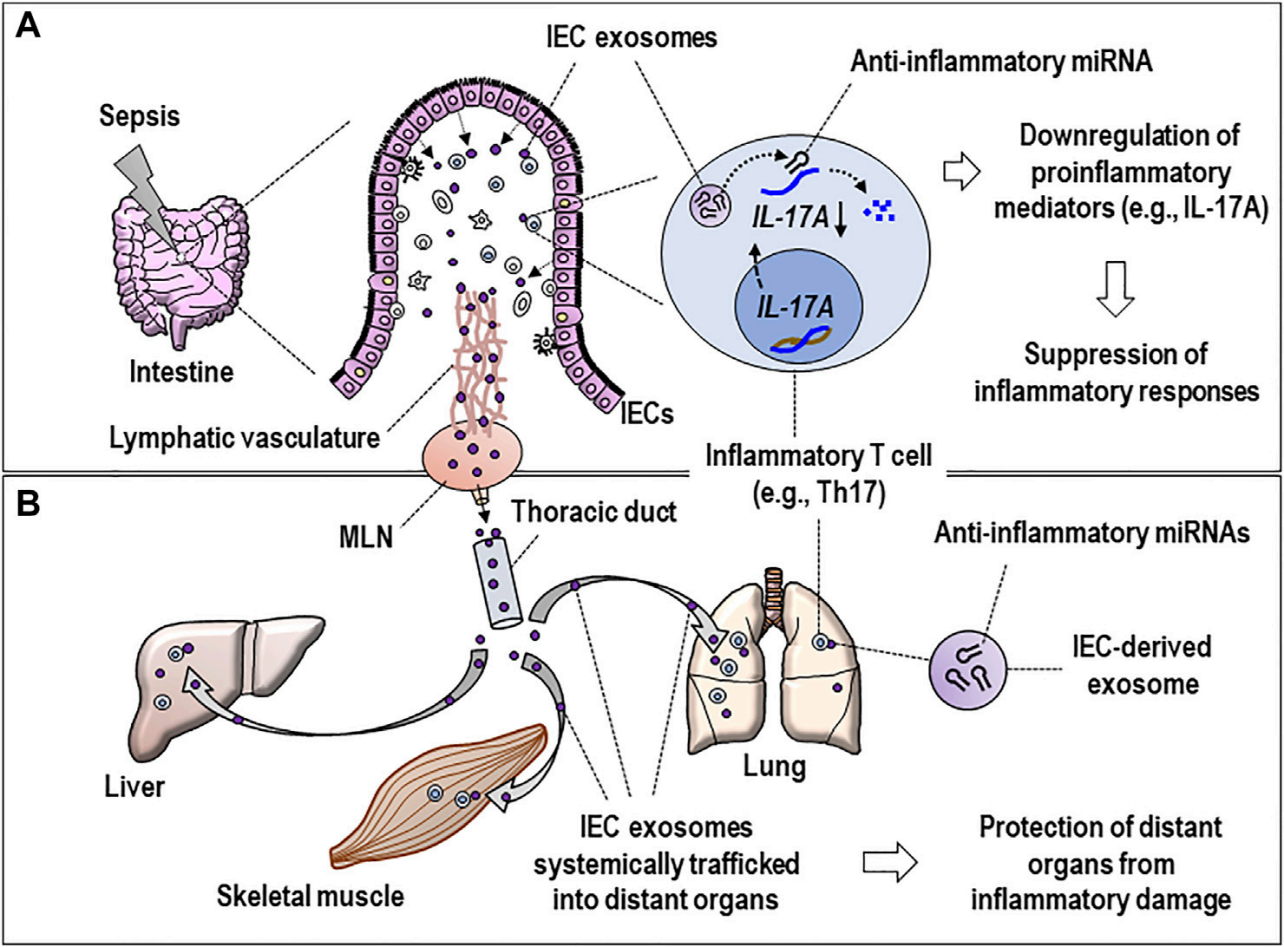
Hypothetical model for the dynamic activity of basolaterally released septic IEC exosomes in regulating inflammatory responses at local and distant tissues. **(A)** IEC exosomes reach inflammatory Th17 cells in the lamina propria of inflamed intestines and deliver miRNAs (e.g., miR-22 or miR-126a, see text) to suppress the *IL-17A* expression, proposing the miRNA-induced suppression of inflammatory responses that occurred at local tissues. **(B)** IEC exosomes possessing regulatory miRNAs are assumed to undergo systemic traffic *via* the lymphatic vasculature, MLNs, and thoracic duct and subsequently reach distant organs (e.g., lung, liver, etc.) to further alter functions of inflammatory T cells in multiple organs by the same method of transferring the miRNAs. Eventually, it can be hypothesized that IEC-derived exosomes may be involved in protecting the tissues from inflammatory damage. IEC, intestinal epithelial cell; MLN, mesenteric lymph node; IL-17A, interleukin 17A; Th17, T helper 17.

The functional polarity is a hallmark of IECs and critical in maintaining intestinal homeostasis (Lee et al., 2008; Klunder et al., 2017) and mediating inflammation (Serrano et al., 2019). IECs face the external environment at the apical side and simultaneously interact with other cells such as lymphocytes or dendritic cells (DCs) and the extracellular matrix in the lamina propria (LP) (Boyd, 2008; Iftekhar and Sigal, 2021). IECs are supposed to secrete exosomes from both sides (Park et al., 2017). Apart from the exosomes released from the apical surface, basolaterally secreted epithelial exosomes are thought to be effective in inducing immune responses. Thus, exosomes taken up by DCs transfer exogenous peptides onto major histocompatibility complex class II (MHC II) of these DCs, thereby eliciting adaptive immune responses of CD4 T cells in the LP region (Mallegol et al., 2007). The basolaterally released exosomes may have a chance to reach inflammatory lymphocytes such as T helper 17 (Th17) cells to transfer distinct miRNAs (e.g., miR-22, miR-126a, etc.), thereby downregulating the expression of *IL-17A* (Figure 2A). This process is thought to contribute to mitigating inflammatory response and damage in the local tissue (Figure 2A).

The basolaterally released IEC exosomes undergo intertissue traffic *via* a systemic circulation and affect septic pathogenesis in distant organs (Park et al., 2019b). Likewise, the exosomes basolaterally secreted by IECs can be feasible to reach distant organs *via* the thoracic duct and systemic circulation (Figure 2B). As a compelling speculation, these IEC exosomes possessing regulatory miRNAs alter functions of inflammatory T cells in multiple organs by miRNA transfer to those cells, which may implicate a recovery process to protect the tissues from further inflammatory injury (Figure 2B). Previous reports support the current speculation for *in vivo* trafficking of IEC exosomes into different organs. Kojima et al. showed that the IEC exosomes were found to enter mesenteric lymph nodes (MLNs) through lymphatic vasculature and further reach distant organs such as the lungs in the study using a trauma/hemorrhagic shock model (Kojima et al., 2018a). Intriguingly, the same researchers have also found that these exosomes are involved in induction of acute lung injury *via* macrophage activation (Kojima et al., 2018b). Accordingly, the MLNs might play a role as an anatomical linchpin to transport the exosomes *via* the intestinal lymphatic vasculature from the intestine to the distant organs affecting the pathologic progression of various diseases (Fanous et al., 2007; Bernier-Latmani and Petrova, 2017). As a plausible speculation, the functional miRNAs of perhaps septic IEC-produced exosomes may contribute to fine-tuning systemic inflammation by regulating the expression of proinflammatory mediators at the post-transcriptional stage (Figure 2B).

On the other hand, an enterocytic infection has been thought to boost IEC secretion of exosomes. Huang et al. reported that the enterovirus A71 infection promoted the IEC secretion of exosomes, and an abrogation of the exosomal pathway increased survival rates of the infected animal (Huang et al., 2020). The fact that whether other types of enteroviruses also exert the effect identical to A71 remains unexplored (Huang et al., 2020). More recently, Xi et al. reported that the fecal microbiota transplantation (FMT)-mediated intestinal microfloral change induced IECs to facilitate secretion of their exosomes in a sepsis model using rats (Xi et al., 2021). Moreover, these IEC exosomes turned out to play a role in M1 polarization of macrophages in MLNs, thereby increasing IL-1β circulation, which consequently elicited neuronal damage and apoptosis (Xi et al., 2021). This study raises a possibility that IEC exosomes function as a systemic messenger to devastate sepsis-associated brain disease, although a question about how these IEC exosomes’ miRNAs functionally associate with developing this neurological illness needs to be addressed.

### Age-Associated Impairment of the Intestinal Barrier or Integrity

Aging raises septic incidence, severity, and mortality (Girard et al., 2005; Martin et al., 2006; Martin-Loeches et al., 2019). Sepsis-induced intestinal barrier dysfunction becomes devastated with age, which might be partly attributed to multiple factors, including oxidative stress, metabolic impairment, antimicrobial peptides, and microbial dysbiosis (Rera et al., 2012; Crapser et al., 2016; Thevaranjan et al., 2017; Nie et al., 2019). In addition, defective bloodstream, ischemic alterations, and the increased use of anti-inflammatory drugs associated with malnutrition and pathogenic microflora are expected to contribute to disruption of epithelial integrity in elderly patients (Meier and Sturm, 2009). Aging itself is thought to increase intestinal permeability and resultant inflammation due to alterations in barrier molecules (Man et al., 2015; Nicoletti, 2015) and microbiota (Thevaranjan et al., 2017). Interestingly, this intestinal barrier dysfunction can be seen even in *Drosophila* with aging (Rera et al., 2012), indicating that this pathologic event is common to various species of aged living organisms. Among the molecules functional in maintaining epithelial integrity, tight junction (TJ) proteins connecting the plasma membrane of IECs at apical sides can be a representative target whose expression is declined by aging (Mitic and Anderson, 1998). Accordingly, aging increases IEC permeability and the subsequent microbial influx through reducing the expression of integral proteins, which also contributes to an age-linked chronic state of inflammation, termed inflammaging (Franceschi and Campisi, 2014; Branca et al., 2019).

### Proposed Roles of Intestinal Epithelial Cell MicroRNAs in Intestinal Permeability

**FIGURE 3:**
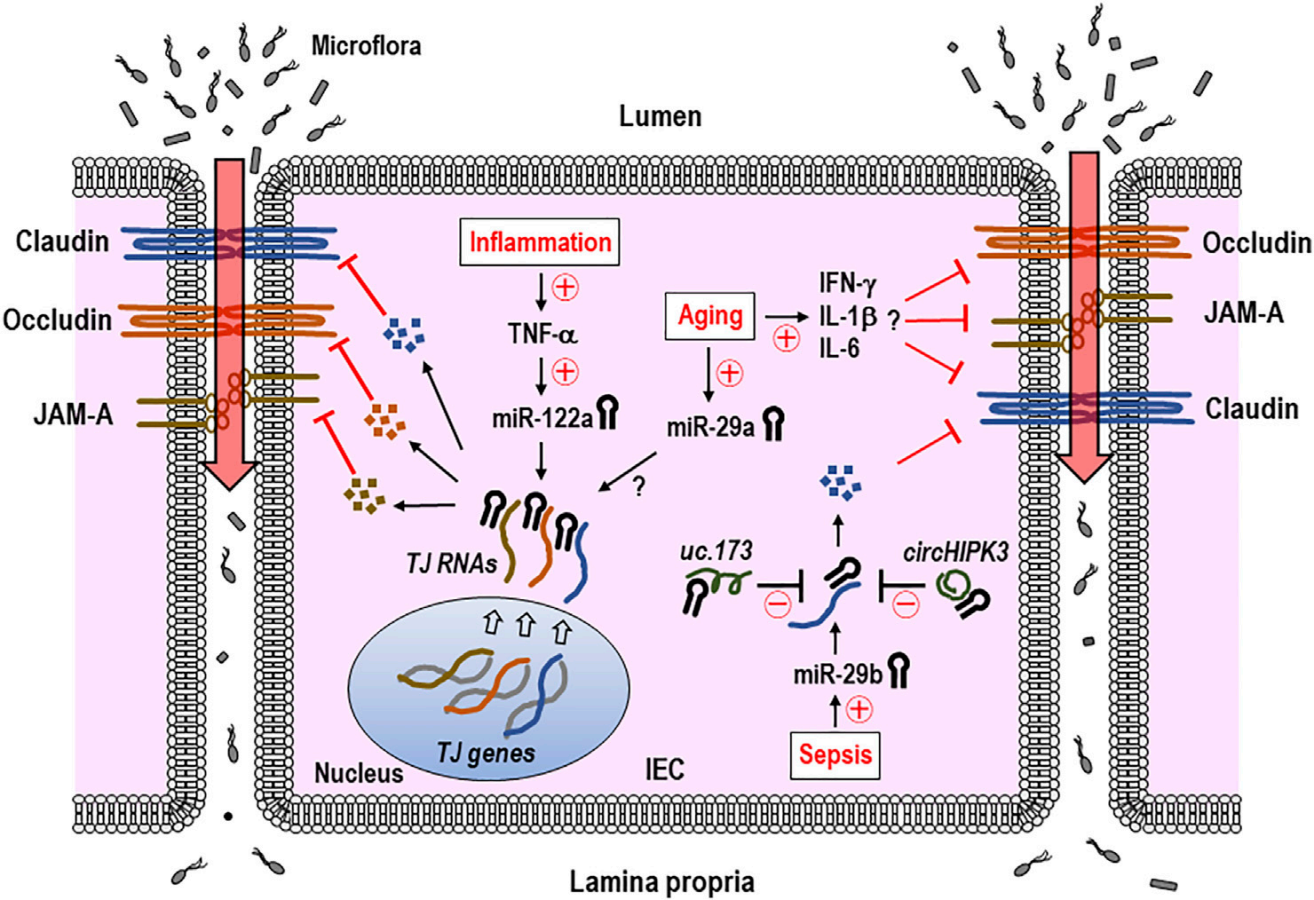
IEC miRNAs regulate the expression of tight junction (TJ) proteins and intestinal permeability in inflammation, aging, or sepsis. TNF-α-induced increase in IEC miR-122a during gut inflammation suppresses the gene expression of TJ proteins including occludin and raises intestinal permeability. Aging-associated elevation of miR-29a and proinflammatory cytokines, such as IFN-γ, IL-6, and IL-1β is thought to downregulate TJ expressions, although it remains unknown that miR-29a directly binds to 3′-UTR of TJ RNA. Some of the non-coding RNAs (ncRNAs), including *uc.173* and *circHIPK3*, were shown to interfere with miR-29b′s function in regulating the TJ expression of claudin-1 by binding to the sites within this miRNA specific for those lncRNAs. Therefore, some of epigenetic gene regulators including miRNAs and ncRNAs appear to engage in regulating and recovering TJ expressions, respectively, and subsequent intestinal epithelial barrier. The miRNA-led reduction of the TJ proteins induces an increase in the intestinal permeability and influx of microflora and their products, which may eventually cause intestinal inflammation. Claudin, occludin, and JAM-A indicate representative TJ proteins. Circled +, increase; circled −, decrease; TJ, tight junction; JAM-A, junctional adhesion molecule A; IEC, intestinal epithelial cell; IFN-γ, interferon γ; IL-1β, interleukin 1β; circHIPK3, circular homeodomain-interacting protein kinase 3.

In this section, we discussed more about the possible roles played by epigenetic regulators (e.g., miRNAs) in remodeling TJ proteins of intestinal epithelia and disrupting the epithelial integrity on intestinal inflammation or aging. Cichon et al. have overviewed the miRNAs engaged in regulating the intercellular permeability in both epithelial and endothelial barriers of different tissues, including the brain (Cichon et al., 2014). Ye et al. have reported that the miR-122a accelerates the intestinal permeability by targeting occludin to downregulate its expression (Ye et al., 2011). In this study, using *in vitro* (Caco-2 cells) and *in vivo* (mice) analyses, disruption of the TJ occludin barrier under the inflammatory condition has proven to be because of the roles played by the IEC miR-122a increased by TNF-α (Ye et al., 2011) (Figure 3). In a model of a non-human primate, baboon, aging was shown to enhance permeability in the large intestine in which the expressions of different TJ molecules, including zonula occludens 1 (ZO-1), occludin, and junctional adhesion molecule A (JAM-A), were downregulated in the biopsy (Tran and Greenwood-Van Meerveld, 2013). Of note is the fact that the increase in miR-29a occurred in the same biopsy along with elevation of proinflammatory cytokines such as IFN-γ, IL-6, and IL-1β (Tran and Greenwood-Van Meerveld, 2013) (Figure 3). It still remains obscure for molecular mechanisms by which miR-29a epigenetically regulates the expression of TJ molecules. Nonetheless, these findings acquired using the non-human primate model would be an imperative addition to the scientific note that the age-driven increase in intestinal permeability arises from remodeling the epithelial TJ expression, which is perhaps induced by miRNAs such as miR-29a.

Long non-coding RNAs (lncRNAs), which are a class of non-coding RNA molecules and >200 nucleotides in length, regulate the gene expression and a variety of cellular processes (Ulitsky and Bartel, 2013). Zou et al. reported the interesting findings of roles played by a lncRNA in regulating intestinal epithelial integrity (Zou et al., 2016). *H19* lncRNA was shown to act as a precursor of miR-675 in the study, in which the *H19* overexpression in epithelial cells enhanced miR-675 to post-transcriptionally downregulate ZO-1 and E-cadherin important for the intestinal barrier function (Zou et al., 2016). Wang and colleagues demonstrated another lncRNA, *uc.173*, played a role in alleviating the gut permeability (Wang et al., 2018). Specifically, debilitation of epithelial integrity in the septic mouse model was relieved by *uc.173* treatment, *via* the role played by *uc.173* in neutralizing the miR-29b that targets TJ claudin-1 (Wang et al., 2018) (Figure 3). Circular RNAs (circRNAs), another class of ncRNAs, are known to possess single or more binding sites for miRNAs (Memczak et al., 2013; Thomas and Saetrom, 2014). Recently, Xiao et al. demonstrated a role of circular RNAs (circRNAs) in ameliorating the intestinal epithelial barrier (Xiao et al., 2021). In this study, using *in vivo* models of sepsis mice and *in vitro* cell lines, circular homeodomain-interacting protein kinase 3 (*circHIPK3*), a circRNA transcribed from the *HIPK3* gene (Zheng et al., 2016), was shown to recover intestinal barrier integrity by binding miR-29b to reduce its function, which was presumably due to the restored expression of its targets such as Rac1, Cdc42, and cyclin B1 (Xiao et al., 2021) (Figure 3). Zou et al. have reported a transcription factor JunD enhances miR-29b levels to interfere with IEC growth (Zou et al., 2015), providing a possibility to treating the intestinal degenerative disorder by exploiting an option of JunD inhibition and subsequent decrease in miR-29b. Eventually, the miRNA-induced increase in intestinal permeability and subsequent influx of microflora and their products cause intestinal inflammation (Figure 3). Collectively, some of the epigenetic gene regulators including miRNAs, lncRNAs, and circRNAs are thought to contribute to controlling the intestinal epithelial barrier.

### Other Studies on Intestinal Epithelial Cell MicroRNAs Predicted to Influence Intestinal Inflammation

With regards to regulatory roles of miRNA to target IECs in a cell type-specific manner, Kwon et al. showed that miR-195 was capable of suppressing the function of Tuft and Paneth cells by targeting double cortin-like kinase 1 (DCLK1) (Kwon et al., 2021). The epithelial miR-195-transgenic mice exhibited the enhanced permeability of intestinal epithelia upon LPS treatment, suggesting that the blockade of this miRNA can be a potential therapeutic option to improve the intestinal integrity (Kwon et al., 2021). In the investigation of miRNA-mRNA network profiles in the IECs of mice with inflammatory bowel disease, the increase in miR-3473a, miR-1224, and miR-5128 was expected to associate with downregulating aquaporin 8, which may induce mucus reduction and barrier loss of IECs (Lee et al., 2015). In another study using aged mice, the activation of key pathways implicated to TLRs and notch signaling was shown in IEC miRNAs in an age-dependent manner (Lee et al., 2021). Specifically, the miRNAs, including let-7, miR-7a, miR-92a, miR-200c, miR-760, miR-1224, miR-5099, and miR-5129, were thought to participate in driving those cascades (Lee et al., 2021). Thus, further examinations on IEC miRNAs and their regulatory pathways will be beneficial in better understanding the molecular mechanisms by which age devastates or the pathophysiological events in the gastrointestinal tissues. Table 1 summarizes IEC- or IEC exosome-derived non-coding RNAs, including miRNAs, lncRNAs, or circRNAs, and their functions discussed in this review.

**TABLE 1:** IEC- or IEC exosome-derived noncoding RNAs and their functions discussed in this review.

Non-coding RNA	Source	Related protein or target	Function	Reference
let-7 family	IEC exosome	TLR4 and SNAP23	Antimicrobial protection to *C. parvum*	Hu et al. (2013)
miR-146b-5p	IEC exosome	KLF4 and cyclin D2	IEC growth inhibition and gut injury in sepsis	Tong et al. (2020)
miR-19a, -21a, -27a, -126a, etc.	IEC exosome	TNF-α and IL-17A	Downregulation of gut inflammation	Appiah et al. (2020)
miR-122a	IEC exosome	TNF-α and occludin	Intestinal barrier dysfunction	Ye et al. (2011)
miR-29a	IEC exosome	ZO-1, occludin, and JAM-A	Intestinal permeability facilitation	Tran and Greenwood-Van Meerveld, (2013)
*H19* (miR-675 precursor)	IEC	ZO-1, E-cadherin	Downregulation of epithelial integrity	Zou et al. (2016)
*uc.173* (miR-29b antagonizing)	IEC	Claudin-1	Rescue of sepsis-induced gut permeability	Wang et al. (2018)
*circHIPK3* (miR-29b antagonizing)	IEC	Rac1, CDC42, and cyclin B1	Increase in intestinal barrier integrity	Zheng et al. (2016)
miR-29b	IEC	JunD	Decrease in intestinal growth and function	Zou et al. (2015)
miR-195	IEC	DCLK1	Increase in intestinal permeability	Kwon et al. (2021)
miR-1224, -3473a, and -5128	IEC	Aquaporin 8	Mucin reduction and IEC barrier loss in IBD	Lee et al. (2015)
let-7, miR-7a, -92a, etc.	IEC	TLRs	Age-related gut inflammation	Lee et al. (2021)

## Conclusion

Due to their polarized morphology and distinct positioning, IECs release the exosomes *via* both apical and basolateral sides. IEC exosomes in sepsis have been thought to affect sepsis-induced multiple organ dysfunction. Paradoxically, the IEC exosomes apically released during sepsis may contain the miRNAs to possibly exhibit an anti-inflammatory ability to the inflamed gut. In this regard, it can be considered that basolaterally released IEC exosomes may systemically migrate to distant organs and play a role in alleviating the septic inflamed tissues, presumably through transfer of regulatory miRNAs into inflammatory cells. Aging devastates, or synergizes more with sepsis, pathologic symptoms including intestinal permeability and subsequent inflammation. The TJ proteins in IECs are the main target molecules of intestinal disintegrity during sepsis and aging. A group of miRNAs expressed in IECs engage in post-transcriptionally regulating the mRNAs of those targets during sepsis, inflammation, and aging, while some of lncRNAs are capable of nullifying the miRNAs’ function by binding to them beforehand. Further investigations of IEC exosomes and miRNAs would provide an insight into developing efficacious drug-delivery systems using biomaterials (e.g., mimics or inhibitors of functional miRNAs and exosome-like nanoparticles, etc.), which would be able to help treat sepsis- and age-associated intestinal disorders in the near future.
